# Game Design to Measure Reflexes and Attention Based on Biofeedback Multi-Sensor Interaction

**DOI:** 10.3390/s150306520

**Published:** 2015-03-17

**Authors:** Inigo de Loyola Ortiz-Vigon Uriarte, Begonya Garcia-Zapirain, Yolanda Garcia-Chimeno

**Affiliations:** Deusto-Tech-LIFE Department, University of Deusto, Bilbao 48007, Spain; E-Mails: mbgarciazapi@deusto.es (B.G.-Z.); yolanda.garcia@deusto.es (Y.G.-C.)

**Keywords:** biofeedback, multi-sensor, game, Kinect, Eye Tracker, GSR, EMG, pulsometer, respirometer

## Abstract

This paper presents a multi-sensor system for implementing biofeedback as a human-computer interaction technique in a game involving driving cars in risky situations. The sensors used are: Eye Tracker, Kinect, pulsometer, respirometer, electromiography (EMG) and galvanic skin resistance (GSR). An algorithm has been designed which gives rise to an interaction logic with the game according to the set of physiological constants obtained from the sensors. The results reflect a 72.333 response to the System Usability Scale (SUS), a significant difference of *p* = 0.026 in GSR values in terms of the difference between the start and end of the game, and an *r* = 0.659 and *p* = 0.008 correlation while playing with the Kinect between the breathing level and the energy and joy factor. All the sensors used had an impact on the end results, whereby none of them should be disregarded in future lines of research, even though it would be interesting to obtain separate breathing values from that of the cardio.

## 1. Introduction

The development of methodologies in human interaction with technology has advanced a great deal over the last few decades in fields such as IT, engineering and even psychology. One of these technologies is biofeedback [1], defined as the ability to control certain physical or biological functions by receiving information about them. The biofeedback technique [2] can be subdivided into two main types: direct and indirect. The direct type refers to conscious body reactions such as deliberate contraction of a muscle, while the indirect type refers to unconscious physiological functions (PF) such as heart rate.

This methodology was initially introduced in the field of medicine and, subsequently, spread to other spheres of activity such as IT and video games, with several studies having been carried out in this area about how to use this new technology so as to improve interaction between people and technology [3].

It is thus possible for an individual to be aware of biological functions that they do not perceive under normal conditions, such as heart rate, blood pressure and skin conductance. The information reaches the individual in the form of visual or auditory stimuli which inform them about the state of a specific physiological function. A graduated scale or battery of lights can be used that light up or switch off according to an increase or decrease in physiological response. Sounds are used on some occasions that change intensity or tone. Biofeedback can be classified as several types, depending on the physiological system about which the individual is informed:

- Responses from the somatic nervous system, essentially via electromyogram.
- Responses from the autonomous nervous system that include blood pressure, heart rate, temperature and stomach pH, among others.
- Response from the central nervous system obtained via electroencephalogram, which detects brain rates (alpha, theta, SM and MU waves).

There are some general principles governing this technique such as the treatment of different health problems (sphincter incontinence, anxiety, insomnia, high blood pressure and migraine) [4]. It could therefore be used to train a user in an educational process that involves learning mind/body relationship skills. Biofeedback enables us to ascertain when we are changing our physiology in the direction desired.

In practising this technique, we might be able to familiarise ourselves with our psycho-physiological reactions associated with pressure (nerves, anxiety) and learn to control them better.

There are some specific applications (see Section 2.1) that use some of the major sensors such as: Eye Tracker (eye movement), Kinect (movement), electrocardiogram (ECG, pulse), respirometer, electroencephalography (EEG, brain activity), electromyography (EMG, electrical impulses in the muscles) galvanic skin response (GSR, skin conductivity), thermometer, photoplethysmogram (PPG, blood flow), hemoencephalography (HEG, oxygen in the blood) and Capnograph (CO_2_ in the airway), *etc*.

In this article the authors propose a game application based on biofeedback techniques to measure PF to enable the player to control their stress level. While the game is being played, the user will have access to a constant display of information obtained by these sensors, therefore obtaining real-time feedback that may enable them to become involved in developing the game.

Ultimately, the purpose of this study was to create an application that allows the subject to control their PF. Initially, the authors thought that a stress game would be the best-case scenario for learning to relax. Although in future versions we could change the type of game or apply the biofeedback technique in other fields (such as for watching a scary movie). Another major improvement in this application is that is converts sensors into wireless sensors.

## 2. Background

### 2.1. Biofeedback

Biofeedback refers to the ability to self-regulate the biological or physiological functions of a person by gaining a greater awareness of them using instruments [5] that may provide information about those same systems (see Section 1).

Several sensors connected to the body are normally required in order to acquire vital signals such as those produced by sweat glands (GSR), heart rate (ECG), muscles (EMG), brain activity (EEG) and body temperature. The information obtained using these sensors is shown to the person in audio or computer graph form, or any other type of feedback.

Biofeedback systems normally have multiple processes that correlate information from different sensors and interpret the resulting values in such a way that a response to the user can be processed. Depending on the system’s purpose, the algorithms used need to be adapted to their specifications [6,7], thus requiring a combination of different skills, meaning that developing a Biofeedback system is a complex task.

Under normal conditions, the person ignores how the parameters that measure many of their physiological constants behave. When using this technique, the patient may modify their own physiological states and control their normal functioning [8]. The aim is for the individual to achieve voluntary control of their own physiological states [9] without the use of instruments.

Some of the pathologies in which good results have been noted are as follows: anxiety [10], mild depression, epilepsy [11], headache [12], to improve concentration when learning, to achieve a state of inner calm, muscle tension, neuromuscular re-education, chronic pain [13], high blood pressure, asthma, circulatory problems and disorder owing to lack of attention in children [14].

There is growing acceptance of biofeedback as a first-class treatment method in view of its proven clinical effectiveness [15]. Apart from its practical application in hospitals and clinics all over the world, thousands of studies into different applications vouch for its success. Previous projects have been developed using interactive platforms in the biofeedback environment, generally in games.

In the case of one of them, this technique was used for muscular rehabilitation [16] by applying both relaxation and contraction therapy. The “Space Invaders” game was played, with the shooting control being activated every time the contraction level exceeded a threshold marked in the calibration. In contrast, if the system was configured in relaxation mode, then the shooting command was activated when the EMG sensor level was below the threshold.

As the study’s conclusion indicates, use of the Biofeedback system helped to improve muscular strength and range of movement, where the experimental group exceeded the group that was not taking part in it by 136%, meaning this could be considered a promising method for future research.

Another project [17] featured five game mechanics by introducing biofeedback data into the game. The physiological sensors used as direct control were breathing, temperature and an EMG on the leg. As for indirectly-controlled sensors, a sphygmomanometer and an ECG were used. These game mechanics involved the size of the lens (GSR and RSP), the speed and height of the jump (ECG and EMG), the length of the flamethrower (GSR and RESP) and in the final scene, the time and the difficulty of the enemy (TEMP and ECG).

A special function was also used which, when using an eye tracker, showed a red circle on the screen corresponding to the place where the user was looking, thus enabling the player to momentarily freeze their opponents.

The results proved highly satisfactory: when asked whether they preferred to play with or without sensors, nine out of 10 players preferred to use the control via physiological sensors. In terms of sensor preference, the most voted one was the Eye Tracker, followed by the respirometer. These results indicate that the players preferred direct control over indirect control, owing to the visible response this provided.

Another study [18] involved comparing the use of different types of biofeedback—implicit and explicit—in which the implicit type was associated with the indirect biofeedback referred to previously and the explicit type with the direct form.

The biofeedback system was implemented in a first-person action game and with breathing (RESP) and electrodermal activity (EDA) sensors used as data entry to the system. When the user became nervy (the EDA level rose), the character shook with greater intensity, and moved and shot faster and with greater recoil. In contrast, when the player was relaxed (low EDA level), the character moved more slowly and shot with greater accuracy.

In terms of RESP data entry, when the player inhaled, the character became slower and more accurate, whereas when they exhaled, the character became faster but pointed with greater difficulty.

The experiment consisted of two phases, the first of which involved the players being unaware of the fact that the game was being controlled by their physiological constants—This being the implicit part. The explicit condition manifested itself in the second phase, in which the players were aware of how the system worked and won.

As a result of the experiment, the RESP source experienced no incidents in the implicit part, whereas some interesting factors were displayed in the explicit part, which showed that several players enjoyed playing with this part of the system, as they achieved a greater level of immersion.

Lastly, another study [19] only focused on indirect biofeedback in a first-person action game. The sensors used in this system were a GSR system and a heart rate. The information received from these sensors affected several game functions such as the speed of movement, audio volume, stealth mode, weapon damage, difficulty of the enemy and a variety of effects in the user interface.

Individuals played in two different modes—A standard one and another one using biometric sensors. The results obtained showed that the audio effects had a significant effect on participants’ physiological information and reactions. Participants were more involved in developing the game in the mode that included biometric sensors, especially when there were sound effects.

### 2.2. Sensors

A biofeedback system needs to obtain and process information about the user’s physiological reactions. To do so, a variety of sensors are needed, each of which is responsible for measuring each of the physiological signals.

Below are listed the different sensors involved in the project. Both direct and indirect Biofeedback sensors will be used. In this study, we could also divide the sensors into two types: sensors used only for biofeedback and sensors that the subject only uses to play without any interaction with the biofeedback. Sensors that can be used to play are as follows:

- Eye Tracker

One of the ways in which the Eye Tracker [20] is used is to help people with physical disabilities and to improve their quality of life, as well as that of their family. Thanks to these, the computer can be controlled using the eyes to write, surf the Internet, play and, even more importantly, communicate with the outside world. The possibility of using the eyes like a computer mouse is now being studied, whereby if a person is able to move their eyes and blink, then they will have a new form of communication [21].

- Kinect (Microsoft Kinect)

Kinect [22] is a device created by Microsoft that was originally designed for the Xbox 360 video game console in order for players to be able to interact naturally and intuitively with the console, so as to ensure that playability would be far more agile and games would require hardly any learning by users in order to gain a command of them. This device comprises three different parts: a depth detection system based on an infrared beam sensor, an RGB video camera and a four-microphone array for voice recognition.

The depth sensor consists of a projector with infrared beams that are invisible to the human eye and do not affect usability of the device, together with a monochrome CMOS sensor which picks up the beams that bounce within the environment, thus forming a 3D scene for the space taken up by the device [23].

The innovations that have continued to be made are as follows:

○ Facial recognition [24]: Enables different parts of the face to be recognised and positioned, monitoring in a similar way to what was already being done with the skeleton.
○ Seated mode: Recognises people who are sitting down by recognising their skeleton and monitoring 10 articulations.
○ Monitoring of the skeleton is supported in the closest mode. The player was hitherto only recognised but there was no monitoring of each of their articulations.

The sensors used for biofeedback are as follows:

- Pulsometer

A pulsometer [25] is an electronic device that is mainly used to measure the heart rate both graphically and digitally (heartbeats per minute). Pulsometers are also known as heart rate monitors. They constitute a simple way of keeping the number of heartbeats within advised limits. For people with heart problems or irregular heartbeats, or who have had a heart attack and started to walk every day, carrying a pulsometer can help them to keep within the number of beats recommended by their doctor [26]. Pulsometers are becoming increasingly complex, accurate, safe and reliable.

- Respirometer

The breathing sensor [25] comprises a belt that is fastened around the area of the thorax or abdomen and is used to measure the user’s breathing. As far as its application in biofeedback systems is concerned, it can be used as both a direct and indirect type owing to the fact that it is a function about which the user is generally unaware, but which they can easily end up controlling once they are aware of it [27].

- Galvanic Skin Response (GSR)

Skin conductance, also known as GSR [28], is a method used to measure electrical conductance of the skin, which varies according to its moisture level. The sweat glands are controlled by the sympathetic nervous system, whereby moments of strong emotion change the electrical resistance of the skin. Skin conductance is used as an indication of psychological or physiological excitation.

There are various medical applications based on skin conductance such as control of epilepsy [29], as sweaty hands may be a sign that an epileptic attack is imminent. It can also be used to detect whether there is some sudomotor malfunction, which can help diagnose diabetes, although this sensor is most used in recognising emotions [30]. A study can be created to recognise emotions based on this GSR sensor together with other different devices. Many biofeedback therapy devices use skin conductance to measure and present an individual’s stress response in order to help the user control their anxiety [31].

- Electromyography (EMG)

EMG [28] is a technique used to assess and record electrical activity produced by the skeletal muscles. EMG is recorded using a medical instrument known as an electromyograph, which detects the action potential that activates the muscle cells. When these are either neurally or electrically activated, the resulting signals can be analysed in order to detect any abnormalities and the activation level, or the biomechanics of a human’s or an animal’s movement can be analysed.

An electromyogram measures the electrical activity of the muscles when they are relaxed and being contracted. EMG signals are used in many clinical and biomedical applications [32]. EMG is used as a diagnostic tool to identify neuromuscular diseases [33] and assess lumbar pain, kinesiology and motor control disorders.

The muscles can be used to control any type of operation (motor, servo, light, *etc*.) or for the muscles themselves to interact with the environment.

### 2.3. Challenges Involved in This Study

In conclusion, the most important challenge faced by the authors was to coordinate all the sensors to make it possible to play with them at the same time. However, we need to be aware that some sensors may have incompatible functions. Another challenge was to apply the biofeedback technique to the game, providing it with different functions. As a general overview, the specific sensors that were used to play (interaction with the game) and enable biofeedback to be used are the ones selected (see Table 1).

**Table 1:** Sensor specification.

	Brand Name	Model	Specification
**Sensor to Play**			
Kinect	Kinect for Windows	Version 1.5	Structured-light. Depth sensor: 1.8 to 3.5 m. IR Depth Image: 320 × 240. Colour image: 640 × 480. No IR. Field of view: 57° (horizontal) and 43° (vertical). Minimum latency: 102 ms.
EyeTracker	Tobii	X1 Light Eye Tracker	Variable sampling rate: 28–32 Hz. Total system latency: 50–90 ms, Time to tracking recovery: immediate (blinks), 100–300 ms (after lost tracking). Freedom of head movement at 65 cm: 44 cm (X), 32 cm (Y). Gaze accuracy: 0.5° (binocular and monocular). Precision: 0.20° (binocular), 0.27° (monocular).
**Sensor to Biofeedback**			
Pulsometer and respirometer	Zephyr	BioHarness 3	HR Range: 25–240 BPM. BR Range: 4–70 BPM. Acc. Range: ±16 g. Rechargeable Lithium Polymer. 26 hours per charge. 300 charge cycles. Transmit Range: to 300 ft, 1000 ft w/Antenna and amplifier. Frequency: 2.4–2.4835 GHz.
EMG and GSR	Arduino	e-Health sensor shield v2.0 for Arduino	8 non-invasive + 1 invasive medical sensors. Monitoring EMG signals. Galvanic skin response measurements. Biometric information 6 connectivity options available: Wi-Fi, 3G, GPRS, Bluetooth, 802.15.4 and ZigBee.

Once we have thus assimilated all biofeedback concepts and sensor functions, we could then proceed to design the application.

## 3. Material and Methods

### 3.1. Questionnaires

The individuals who took part in the study were required to complete some questionnaires both before and after. In this way, we can evaluate the game, the interaction with sensors and the comfort factor attached to each sensor (see Supplementary Information).

The first of them was a socio-demographic questionnaire, in which they were asked for their age, sex, ocular condition, whether they use glasses or lens when carrying out the trial, whether they use games and how often they do so, and the type of games. It thus collects the relevant information required about each subject.

Once the individual had completed the trial, they had to fill in a further three questionnaires. One of them was a Perceived Stress Scale (PSS) one [34,35], which measures certain stress-related factors.

The other two were related to the usability of and the individual’s satisfaction with the tool. The System Usability Scale (SUS) [36] was chosen for usability, whereby the tool itself is rated on a technical level. Lastly, they were made to complete some items in which they had to rate the sensors’ comfort, their preferences and the type of game they liked the most (Eye Tracker or Kinect).

### 3.2. Participants

Fifteen individuals were selected for this project. The descriptive features of these participants are shown in Table 2.

**Table 2:** Socio-demographic parameters of the participants in the study.

	f (%) *	Max	Min
**Age [$\bar{\text{X}}$(S.D.)]**	26.47 (3.357)	33	22
**Sex**			
Male	66.70%		
Female	33.30%		
**Ocular condition**			
Yes	60.00%		
No	40.00%		
**Glasses**			
Yes	26.70%		
No	73.30%		
**Contact Lenses**			
Yes	20.00%		
No	80.00%		
**Videogame frequency**			
No	26.70%		
Sometimes	40.00%		
Weekend	20.00%		
3-4 days	6.70%		
Every day	6.70%		

* valid percentage.

This table shows the mean, standard deviation and maximum and minimum age of the participants. Other parameters used are sex, ocular condition, whether they use glasses or lenses at the time of the study and how often they use video games.

Regarding ocular condition, nine of the participants had some eye defect: seven were short-sighted, one was long-sighted and one was long-sighted with a squint.

The video games most commonly used by participants among the 11 who often play them are as follows: 45.46% played simulation games, 18.9% played arcade games and 18.9% online board games.

It should also be mentioned that when carrying out the trial, not all were done in the same way by all the users, *i.e.*, participants were selected randomly (randomly with women on the one hand and on the other with men, thus ensuring that not all women performed them in the same way or vice-versa), so that some of them would first play with the Kinect and then with the Eye Tracker, and the others would do so the other way round. This prevented the tendency for participants to feel uncomfortable with the first game mode owing to lack of knowledge about it and so that they would control the tool better for the second one. Thus, the Kinect would not always be the first one and the Eye Tracker not only the second one.

Therefore, of the 15 participants, six of them (three men) first carried out the trial with the Eye Tracker and subsequently with the Kinect, while the nine remaining participants (seven men) first played with the Kinect and then with the Eye Tracker.

In Table 3, the mean, standard deviation and maximum and minimum from the PSS questionnaire can also be noted. In the case of the PSS, the total score from the questionnaire is obtained together with the six factors referred to, which are listed by name in the table itself.

**Table 3:** Results of PSS questionnaire (mean, standard deviation, maximum and minimum).

PSS	[$\bar{\text{X}}$(S.D.)] *	Max	Min
Total	0.3141 (0.14238)	0.72	0.10
F1 (Tension-fatigue)	0.3284 (0.17644)	0.81	0.11
F2 (Conflict-social acceptance)	0.1683 (0.14042)	0.62	0.00
F3 (Energy-joy)	0.3778 (0.19135)	0.73	0.07
F4 (Overload-harassment)	0.4278 (0.17499)	0.83	0.17
F5 (Self-fulfillment-satisfaction)	0.3481 (0.21359)	0.78	0.00
F6 (Fear-anxiety)	0.3222 (0.21331)	0.83	0.00

* values from 0 to 1.

In the case of sensor values obtained while the tool is being used, the mean, typical deviation and maximum and minimum are shown in Table 4 and Table 5. Apart from the values obtained from each sensor at the start of the trial, at the end and the maximum and minimum values during the trial, the differences between the starting and end values (start-end) and maximum and minimum values (maximum-minimum) have also been included.

**Table 4:** Mean, maximum and minimum results of the physiological values in the Eye Tracker mode.

Eye Tracker	[$\bar{\text{X}}$(S.D.)]	Max	Min
Initial Cardio	70.20 (11.060)	87	51
End Cardio	72.33 (14.266)	100	50
Maximum Cardio	79.53 (11.476)	100	62
Minimum Cardio	66.40 (10.432)	81	50
Difference Cardio Time (End-Initial)	2.13 (7.549)	−6	19
Difference Cardio Peak (Max-Min)	13.13 (3.461)	9	19
Initial Breathing	10.40 (4.421)	20	3
End Breathing	11.00 (4.408)	19	6
Maximum Breathing	13.73 (3.327)	20	9
Minimum Breathing	7.93 (3.595)	15	3
Difference Breathing Time (End-Initial)	0.60 (3.738)	−5	6
Difference Breathing Peak (Max-Min)	5.80 (1.146)	4	8
Initial GSR	153.93 (37.351)	219	107
End GSR	164.47 (43.602)	231	101
Maximum GSR	169.20 (38.693)	231	116
Minimum GSR	152.27 (40.229)	212	101
Difference GSR Time (End-Initial)	10.53 (16.331)	−14	48
Difference GSR Peak (Max-Min)	16.93 (18.949)	−23	48

**Table 5:** Mean, maximum and minimum results of the physiological values in the Kinect mode.

Kinect	[$\bar{\text{X}}$(S.D.)]	Max	Min
Initial Cardio	73.60 (11.599)	90	56
End Cardio	73.80 (12.907)	99	52
Maximum Cardio	80.13 (11.096)	99	63
Minimum Cardio	68.93 (11.094)	84	52
Difference Cardio Time (End-Initial)	0.20 (6.657)	−15	11
Difference Cardio Peak (Max-Min)	11.20 (3.189)	8	18
Initial Breathing	10.87 (4.998)	22	6
End Breathing	9.73 (4.317)	20	4
Maximum Breathing	13.60 (3.961)	22	8
Minimum Breathing	7.40 (2.772)	14	4
Difference Breathing Time (End-Initial)	−1.13 (4.749)	−8	8
Difference Breathing Peak (Max-Min)	6.20 (1.781)	3	10

### 3.3. Sensors

The process carried out to develop the sensors is explained during this stage. Selection and connection of the tool to the sensors are taken into account in this part.

#### 3.3.1. Sensor Selection

The user may select the sensors with which they are going to interact while the tool is being used and from which data is to be gathered for subsequent analysis of the aforementioned sensors. To do so, the different incompatibilities between the sensors that can be selected are taken into account.

To this end, there are two types of game: one uses the Kinect sensor as the main one and the other the Eye Tracker sensor. It is also possible to choose sensors which are compatible with them, depending on which type of game one wishes to select. This is due to the fact that there are sensors which are sensitive to user movements (EMG and GSR), meaning they are incompatible with the Kinect sensor. In such case, the sensor that measure the pulse and breathing are the ones that can be selected. Unlike in Kinect sensor mode, the Eye Tracker mode is compatible with others mentioned above such as the GSR and EMG.

Furthermore, to be able to select these sensors, they will need to be connected as otherwise the tool fails to recognise them, not allowing the user to select it. To this end, the user is warned via a message indicating which sensors are connected and which ones are not. If they are connected at that moment, it will be possible to refresh the tool by pressing a button that once again shows the availability of the sensor, enabling it to be selected.

#### 3.3.2. Sensor Connection

An initial attempt is made to connect the sensors with the tool to ascertain whether it is available for possible selection by the user. This occurs once the application is in the window in which the sensors can be selected and every time the refresh button is pressed (see Section 3.3.1).

All the sensors used in this tool are connected via COM ports, although in the case of the pulse and breathing sensor, the same COM port is used (as the same sensor performs both functions).

The user will need to select the sensors they wish to use prior to starting the game, and so only those deemed necessary will be connected. In the case of the sensor that gathers data about the pulse and breathing, communication is via Bluetooth technology [37] via the COM port. The tool used will be the one that starts the communication, indicating exactly what one wishes to receive, and then the sensor will start to send the relevant data.

The Zephyr BioHarness [38] tool sends to be able to receive the General Data Packet (GDP) links which contains the data required. The data packet containing the specific values to enable it to be sent to the sensor will need to be formed.

The Eye Tracker sensor will also be connected via a COM port using the specific library so as to be able to connect to that sensor [39]. The tool mainly checks to see whether the Eye Tracker is connected. Once the identifying number for this sensor has been connected, the connection is then made using the library. The sensor is kept waiting until such time as the tool requires the data, whereby the data required will start to be transmitted.

In the case of the Kinect sensor, which is also connected via the COM port, it is checked to see whether it is ready to receive so that once the tool indicates this, it starts to emit data. Lastly, in the case of the Arduino [40] sensor that contains the GSR and EMG sensors, the connection is opened with the port to which they are connected and, in the same way as with the Zephyr sensor, communication will also be via data packets.

#### 3.3.3. Sensors that the Biofeedback Technique Provides

The specific nature of this technique (see Section 2.1) is based on the feedback of data related to PF. Therefore, depending on the data gathered by the sensors, the game designed for this tool will evolve differently, which is why the user will need to be able to perceive these changes so as to try and learn how to consciously change them via their constants.

The functions offered by the tool with sensors are varied, and the former may be chosen by the user so that they may have a bearing on play in a different way each time. For pulse, breathing and GSR sensors, the functions that exist that may have a bearing on the evolution of play are the same, although the same function cannot be in two sensors at the same time. The functions that can be assigned to these sensors so as to influence play are: the change in speed depending on constants, the appearance of a greater number of cars, the more frequent appearance of oil stains on the tarmac, the more frequent appearance of wheels and the more frequent appearance of petrol drums.

In contrast, in the case of the EMG sensors, although the functions would seem to be similar, the end result of the action taken by the user changes. If the speed function is assigned, the car will go faster for a specific period of time when the muscle is contracted. As for the other functions, in the case of cars or oil stains, the oil stain is covered with a drop of water or the car moves over to the kerb when the muscle is flexed. If the wheels or petrol drums appear, contracting the muscle will influence the appearance of a new drum or a new wheel.

### 3.4. Game

This section describes how the game is played in both modes. Firstly will be explained the evolution of the game itself, then how the data is obtained and finally the biofeedback included in the tool.

A brief explanation firstly has to be given as to what each mode of play available in the tool consists of. One of the modes involves body movement, more specifically of the hands, and the sensor that enables this type of detection to be made is the Kinect. The other mode of play is handled with the eyes, and the Eye Tracker sensor is the one used for taking into account the position of eye gaze on the screen.

Once the mode of play and the sensors compatible with each mode have been selected, the function that one wishes to give to each sensor while the game is being played can then be chosen, which will influence the process to ensure that the technique known as biofeedback takes place (see Table 6 and Table 7). If no function has been selected for any of the sensors that have been previously selected, the user will be warned that the biofeedback function will not be activated, although the data obtained while the game is being played will still be gathered.

**Table 6:** Sensor functions for the Eye Tracker mode.

SENSOR	FUNCTION
Pulsometer	Car speed
Breathing	N cars
GSR	N fuel drums
EMG	Remove oil stain

**Table 7:** Sensor function for the Kinect mode.

SENSOR	FUNCTION
Pulsometer	Car speed
Breathing	N cars

**Figure 1:**
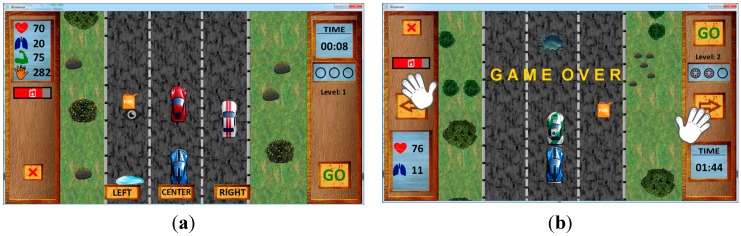
Game interface. (**a**) Eye Tracker mode; (**b**) Kinect mode.

Once everything necessary has been selected, the game will start to be played in the mode selected with the physiological sensors, which involves taking control of a car that proceeds along a three-lane carriageway (see Figure 1). As the car proceeds along this road, other cars appear on the lanes that the user will have to avoid so as not to collide with them, as the game will come to an end if this occurs. Oil stains will also appear on the tarmac that will have to be avoided like the other cars, although if they are driven over, the movements when changing lane will be slower for a period of time.

Apart from the obstacles to be avoided, the game features different levels of difficulty which will steadily increase when the car controlled by the user obtains wheels that appear as the game progresses. Every time three wheels are obtained, the user proceeds to the next level. The difficulty increases proportionally as the level gets higher, increasing the speed of the car.

Another additional difficulty is the number of cars that appear as obstacles, as the number of cars will increase as the level gets higher. A further difficulty with the game is the cost of petrol. As the car proceeds, the petrol tank constantly gets emptier. The game comes to an end once the tank is empty. To ensure this does not happen, petrol drums appear which will completely fill the tank once collected.

Depending on the mode of play that has been chosen previously, small modifications are made to the interface to make the game more comfortable for each sensor. Two hands are shown in the Kinect sensor mode which will serve as a guide for the user to know where they are both located. Two buttons will appear on the screen to enable the car to be moved, each of which is indicated with arrows for the user to press in order to change lane.

Additionally, in the mode of play with the Eye Tracker sensor, the image of an eye will appear instead of hands to indicate the point where the user has to fix their gaze. In this case, there are three buttons used to change lane and these are located on the lower part of the interface, each one in each lane. What the user has to do is look at one of the buttons in order for the car to change lane.

### 3.5. Obtaining Data

The tool is ready for data to be displayed on the screen after being processed while the user is playing and at the same time are saved in files if one wishes to display them later. Therefore, data about the game is gathered at the same time as the game is being played, displayed and saved. The data shown ensures that the user is aware at all times of their constants so that they are able influence these at their convenience in order to advance in the game.

### 3.6. Data Processing

There are two different phases in this section which will be explained below. The first explains the processing of data about the sensors and the second describes the subsequent saving of that data for future analysis.

#### 3.6.1. Data Acquisition

First of all, Kinect and Eye Tracker sensors are used to interact with the game, whereby the data received from these sensors will be used to control the game.

Values from the X and Y axes of each eye were obtained in the case of Eye Tracker. From these four values, a mean for X and another for Y were then obtained, and then with the eyes values, the application prints in the interface that the subject is looking at.

In the case of the Kinect sensor, data is only processedto show the hand movement needed to control the game, and consequently, to enable the game to be played. To do so, the user skeleton is obtained and the whole body except the hands is discriminated.

**Figure 2:**
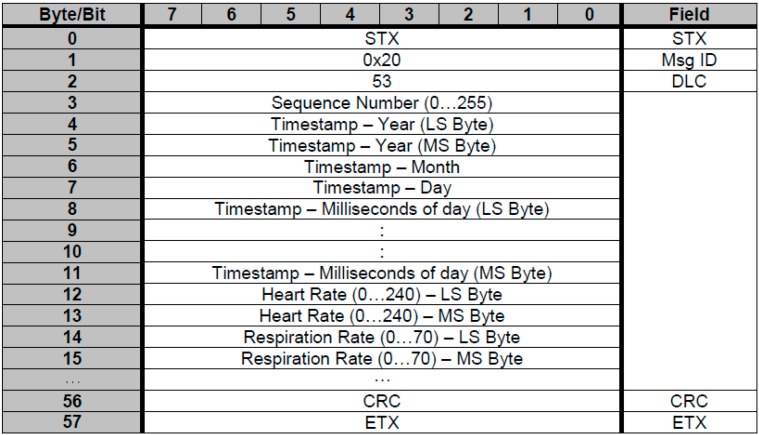
General Data Packet (Pulsometer and respirometer).

In the case of the pulsometer and respirometer sensor, the application receives a General Data Packet (GDP) in which we obtain pulse and breathing values (see Figure 2). Once all the data has been received, we only process that with positions 12 and 14—Heart rate and breathing respectively.

Lastly, in the case of EMG and GSR sensors, we also receive a data packet containing all the values, although the heading is compared so as to differentiate between these two sensors. If the heading starts with “A0”, this refers to EMG sensor, whereas if it starts with “A2”, it will be the GSR sensor.

Specifically, in the case of the GSR sensor, the conductance value requires a change, because the value that we receive does not correspond to the real value, and so a modifications is required to show the conductance (see Equation (1)).

(1) $$conductance\;=2*\left(\frac{voltage-0.5}{100000}\right)$$

#### 3.6.2. Processing of Data Obtained

This processing of the data obtained by the sensors is necessary as the sensors emit data packets which are crude, also sending data of interest such as the user’s vital constants, and sending data that is not so relevant or even dummy data.

Once the data packets sent by each sensor have been made known, those of interest will then be selected and the remainder disregarded. Data of interest is subsequently selected from each data packet, as there is also data to be disregarded in each data packet.

In the case of the Eye Tracker, after carrying out the aforementioned processing, the values for axes X and Y are then selected from each eye. In addition, these values are then checked to see if they are correct and a mean for the X axis of both eyes is calculated together with another for the Y axis of both eyes. These two values that are finally obtained—one for the X axis and one for the Y axis—are the ones that will be used.

In the case of the Kinect sensor, the data is not processed so as to be later saved, but rather, this is done to be able to show the hands on the game screen and thus help the user play the game. To this end, the user’s “skeleton” is obtained which is positioned in front of the sensor camera. The whole body is differentiated from this skeleton except for the positions of both hands.

In the case of the pulse sensor and the breathing sensor, it is the same sensor (see Section 2.2) which measures both user constants. From this sensor, the data packets of interest are the GDP and ECG. For the former, a whole data packet is received from which pulse and breathing values must be obtained that are to be found in specific positions within the data packet, with the remainder being disregarded. For the latter, the values are taken from the data packet from one value to another.

For sensors that measure galvanic skin response and assess and record muscle activity or EMG, data is processed via connection to the Arduino plate. This plate receives the values of the constants from the sensors connected to the e-Health Sensor Platform. The plate then sends the data in hexadecimal with a separate heading for each sensor, and this data will need to be transformed so as to save and show the values obtained from both sensors.

#### 3.6.3. Saving Data

After processing of the data obtained from the sensors, the data is then saved in this second phase. The type of archive where it is saved will be different depending on the sensor used. For instance, in the case of the Eye Tracker, data is saved in an XML file, whereas in the case of physiological sensors, the values obtained are saved in separate TXT files. These files are saved in the folder created for each user when the specific tool was first used.

### 3.7. Data Display

Data can be displayed in two different ways. One of them is separate from operation of the tool and the other involves real-time display of the sensor values while the game is being played.

The real-time display part is shown in the tool itself. On this occasion, the data obtained from the sensors is shown on the tool interface itself as soon as it has been received. If one wishes to display the data once the game is over, then the Matlab tool will be used.

Those files that contain the data one wishes to show will need to be indicated, and the tool will show the results in separate windows.

### 3.8. Statistical Analysis

The SPSS tool was used for all statistical analysis of the results obtained from this study (IBM Corp. Released 2011. IBM SPSS Statistics for Windows, Version 20.0. Armonk, NY: IBM Corp).

The mean, standard deviation and maximum and minimum values for the descriptive results and from the sensors themselves were calculated. The non-parametric Kruskal-Wallis test [41] was carried out on the differences between the initial and final values provided by the sensor at the start and end of the game. The Mann-Whitney test [42] was also carried out so as to be able to ascertain whether there were any significant differences between men and women. No multiple comparisons were made. Correlations were analysed using the Spearman Rank-order correlation.

## 4. Design

Design of this project is divided into high and low level. The former schematically provides a general description of the functions to make it easy to understand the main blocks that comprise the project. The specific functions are described for the low-level design in a more descriptive way. Therefore, everything that has not been able to be included in the high-level design section can be described.

### 4.1. High-Level Design

**Figure 3:**
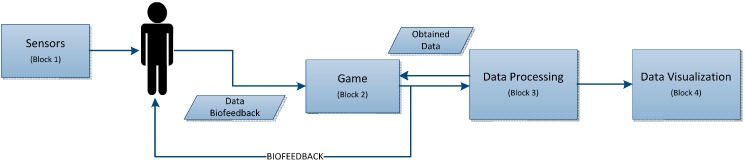
High-level design.

The project is divided into four main blocks. Firstly, there is the block of sensors that send the data received by the latter to the second block, which is the game in which this data is received. Lastly, the processing block and the block for display of the data obtained by the sensors while the game is being played can be seen. The data that can be processed and subsequently displayed refers to everything that directly intervenes in the game being played. Figure 3 shows the block diagram for the high-level design.

#### 4.1.1. Block 1: Sensors

This first block gathers together all the user constants while the tool is being used, with all the data obtained throughout the process being obtained at its output.

This data at the sensor output is needed for subsequent processing and display.

#### 4.1.2. Block 2: Game

This block receives data from the sensors which act in the game in such a way as to modify it according to the data sent from the sensors. A study was carried out to define which of them may interact with the game within the set of all the sensors that intervene in the tool, with those that are able to act most actively either to make the state of play more difficult or to be able to progress being a requirement.

#### 4.1.3. Block 3: Data Processing

In this block can be seen the entry of data sent by the sensor block, which comprises the signals they send. These signals are thus processed in this block in order for them to be possibly displayed.

Lastly, the data gathered by these sensors will be saved for subsequent processing and display.

#### 4.1.4. Block 4: Data Display

In terms of display, the data will be displayed in the simplest possible way to enable it to be interpreted, either via graphs or maps, *etc*., and depending on the data provided by each sensor.

There are two different types of data: that which is only displayed once the tool has finished being used, and that which, apart from being displayed, interacts directly with the game, ensuring that the game’s evolution is modified (speed, number of obstacles, *etc*.).

### 4.2. Low-Level Design

**Figure 4:**
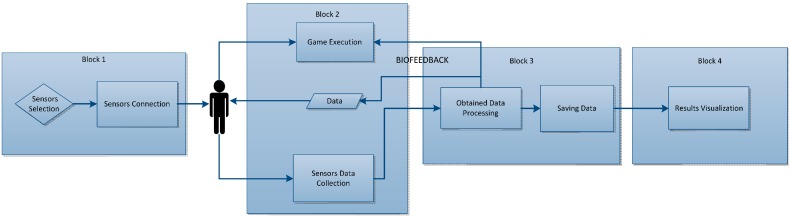
Low-level design.

The functions of the tool are described with greater accuracy in this section. A more exhaustive description of the tool can be given thanks to this type of design. To this end, an organisation chart is shown in Figure 4 below to help proper understanding of the project process.

Once the sensors have been selected, the relevant connections are made to the sensors selected in the previous phase. The next step is to play the game itself, while at the same time gathering the data emitted by the sensors. If the user selects some of this data, they will be able to interact directly as the game evolves, either making it more difficult or easier. This function gives the chance for the Biofeedback technique to be added, as all progress made may be modified if the sensors act in the game process. Lastly and in addition to the biofeedback technique, the data provided by the sensors can be saved after being processed, so as to finally be displayed.

#### 4.2.1. Block 1: Sensors Selection and Connection

**Figure 5:**
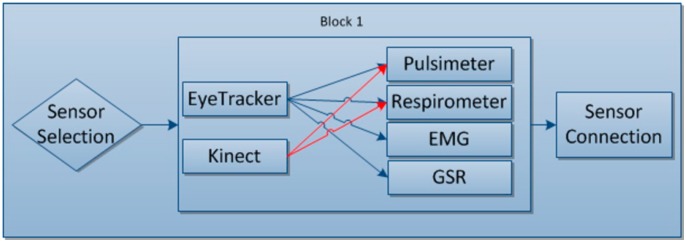
Block 1: Sensor selection and connection.

The user will be able to select the sensors with which they are going to play the game, while always taking into account any incompatibilities there may be among them. Once selected, the connections are made individually as each sensor requires a specific connection. In this block is shown how the sensors can be selected. For the Eye Tracker mode pulsometer, respirometer, GSR and EMG can be chosen and for Kinect mode, because of incompatibilities, only ECG and respirometer as can be seen in Figure 5.

Once the sensors have been selected, they will then each be connected, as each sensor requires a different way to create the connection so as to be able to receive data.

#### 4.2.2. Block 2: Game

**Figure 6:**
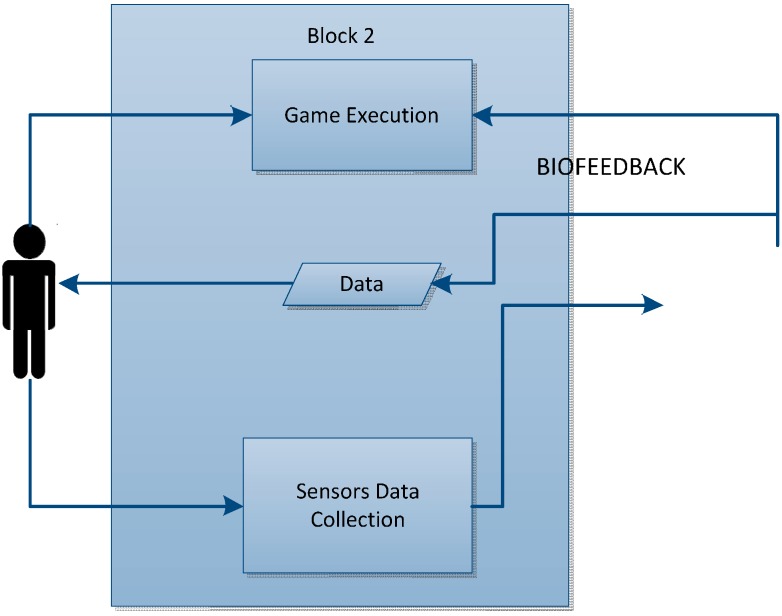
Block 2: Game.

While the game is being played, the data is being gathered from the sensors that are being used by the user. However, in addition to saving data for subsequent display, some data will interact directly with the game. For instance, the game will evolve differently depending on the user’s pulse. This is the biofeedback technique, in which sensors interact with the game in real time, and this can be seen in Figure 6.

The data obtained from the sensors that interact with the game are processed by block 3, meaning there is feedback between the blocks.

#### 4.2.3. Block 3: Data Processing

**Figure 7:**
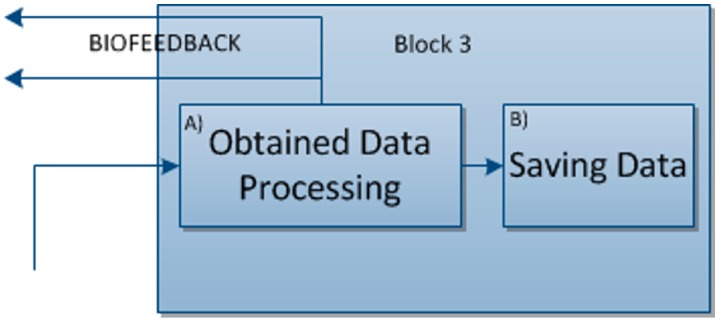
Block 3: Processing and saving data.

Figure 7 shows the block corresponding to the processing and saving of data, which is of special note in that the data processed from block 2 now returns to a suitable format to enable it to interact with the game.

**Figure 8:**
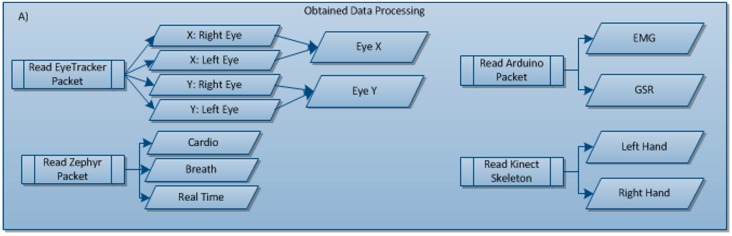
Sub-block for obtained data processing.

In Figure 8, apart from the Eye Tracker and the Zephyr, a data packet will be created to enable data to be obtained from the Arduino sensor, which will offer EMG and GSR data. In contrast, in the case of the Kinect, values are received from the skeleton, which indicates the body’s position at all times. The only thing of interest from this sensor is the position of the user’s two hands to enable them to press the game buttons.

#### 4.2.4. Block 4: Data Display

**Figure 9:**
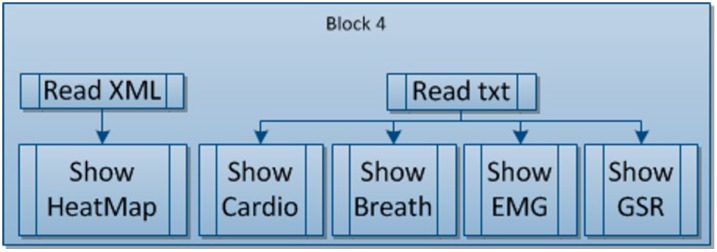
Block 4: Data display.

The results will be able to be shown once the game is over and also while the tool is being used (see Figure 9). If the results are shown once the game is over, this will be on the interface in the most suitable way possible.

## 5. Results and Discussion

Before starting with the results, mention should be made of the fact that the same sensors and the same functions were applied to all participants. The difference lies in the order of modes of play. Table 6 and Table 7 show which functions were assigned to which sensor for both modes of play.

**Figure 10:**
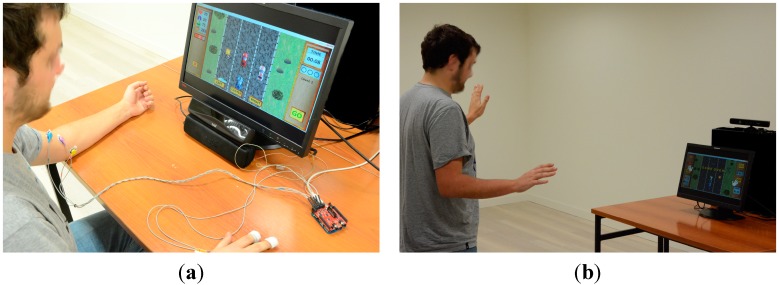
Example of a user playing. (**a**) Eye Tracker mode; (**b**) Kinect mode.

Participants were also subject to a study in a completely isolated room (Figure 10) to ensure there were no distractions.

Those who were going to first play with the Kinect and then with the Eye Tracker and *vice-versa* were selected at random. First, they were explained in which study they were going to take part, being required to sign a consent form for the results to be used. Once the consent form was signed, they were then provided with a socio-demographic questionnaire in order to obtain certain data about the participants.

**Figure 11:**
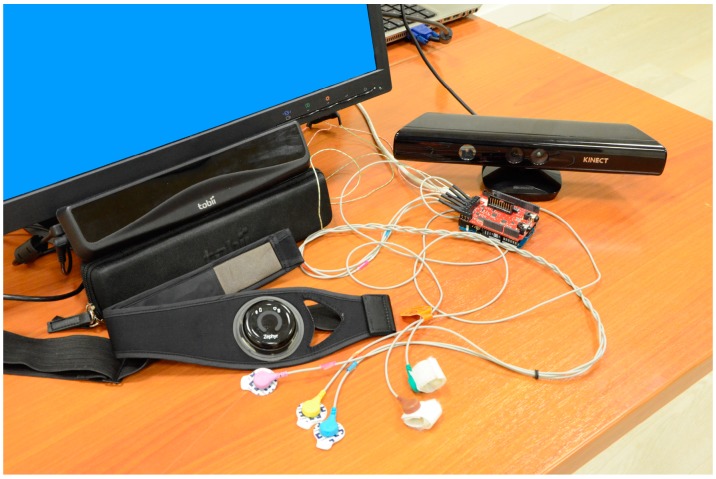
Sensors used in the game.

It was then explained to them which sensors they were going to use with each mode of play and then they were helped with fitting. For the mode of play with Kinect, they were simply going to use the band in which the pulsometer and respirometer sensor comes, and in the case of the Eye Tracker, they were also fitted with the EMG and GSR sensor as well as this band. All sensors can be shown in Figure 11.

They were allowed 2 min for each game, whereby if they lost, they would be able to start again until time ran out. During the game, participants needed further, more explicit instructions so as to be able play properly as, although all functions were explained to them prior to starting, they did not remember all of them or parts that they thought they had understood but that subsequently they had doubts about.

Once the two modes of play had been completed, participants were then provided with the PSS and SUS questionnaire and a further one to rate their satisfaction with the sensors.

### 5.1. Usability and Satisfaction

To measure the participants’ usability and satisfaction with the tool, they had to complete the SUS usability test in addition to certain items referring to sensors.

Table 8 shows the mean score obtained from the SUS questionnaire and the mean obtained from analysis of the sensors when fitting them, the comfort level while the game was being played and the level of control they were able to end up having over the game. This analysis was carried out for each mode of play, *i.e.*, for the Kinect and the Eye Tracker.

**Table 8:** Results of the SUS questionnaire and sensor analysis of user satisfaction.

	[$\bar{\text{X}}$(S.D.)]	Max	Min
**SUS**	72.333 (12.4809)	92.5	45.0
Sensor analysis *			
**EYE TRACKER**			
**Band**			
Fitting	2.87 (1.125)	4	0
Comfort	3.53 (0.516)	4	3
Control cardio	1.60 (1.121)	3	0
Control breathing	1.60 (1.121)	3	0
**GSR**			
Fitting	3.80 (0.561)	4	2
Comfort	3.47 (1.125)	4	0
Control	0.87 (1.246)	4	0
**EMG**			
Fitting	3.27 (0.799)	4	2
Comfort	3.20 (0.862)	4	1
Control	3.00 (1.414)	4	0
**KINECT**			
**Band**			
Fitting	3.07 (1.163)	4	0
Comfort	3.67 (0.488)	4	3
Control cardio	1.87 (1.187)	3	0
Control breathing	1.67 (1.234)	3	0

* the scale ranges from 0 to 4.

As regards the score obtained from the SUS, a mean of 72,333 was obtained from all participants, which can be considered good on the scale of scores provided by the questionnaire and taking into account the fact a minimum score of 68 would be deemed acceptable for a tool [43]. This questionnaire is also a highly-regarded assessment tool, being both robust and reliable, as there is correlation between subjective usability measures.

### 5.2. Sensor Preferences and Type of Game

Participants were asked about certain items (see Section 3.1) so as to ascertain their preferences regarding sensors and the type of game they liked the most. 

Regarding the mode of play with the Eye Tracker, the sensor preferred by most participants was the EMG (53.33%) [17], the second most popular was the pulsometer sensor (66.67%), the third was the breathing sensor (60%), and the least popular among most participants was the GSR (86.67).

The major reasons why participants liked the EMG sense the most were that it is: “easy to interact”, “intuitive” and “very controllable”. Conversely, the main reason why participants did not like the GSR sensor was that: “it is not controllable at all”.

When changing mode of play to the Kinect, the sensor preferred by most participants was the pulsometer (73.33%), whereas the least popular one in this mode of play was the respirometer (73.33%).

These results coincide with the previous ones obtained using the Eye Tracker, as the pulsometer sensor was more highly rated than the breathing sensor. The main reason why participants preferred the pulsometer sensor was that: “the change in car speed can be noted according to the pulse”. The reason why participants did not prefer the breathing sensor was that: “the change in play is less noticeable”.

In terms of mode of play, participants had to respond to the item “I liked playing with the Eye Tracker/Kinect”, providing 5 scores, with the lowest being in total disagreement and the highest being in total agreement.

As for play with the Eye Tracker, 73.33% of participants were in total agreement with the fact they liked this mode of play, 13.33% were in agreement and 13.33% were undecided.

The main reasons why participants liked the Eye Tracker more were that it is: “original”, “a new feature” and “fun to be able to control a game just by looking at it”. In the case of the mode of play using the Kinect, 46.67% were in total agreement, 26.67% were undecided and 6.67” were in total disagreement.

The reason why participants liked this mode of play was: “because they know more about this type of game”. Lastly, participants were asked as to which sensor they preferred out of the Kinect or the Eye Tracker, and 93.33% said they preferred the Eye Tracker.

### 5.3. Inferential Analysis

#### 5.3.1. Kruskal-Wallis Test

Only one significant difference was found between the initial value just at the start of the game and the final value at the end of it from the GSR sensor, when playing with the Eye Tracker (*p* = 0.026).

The reason for this significance is that when this sensor measured stress, anxiety and emotional state, participants underwent a change in their emotional state while they were playing. This is logical, as not wanting to lose and wanting to progress in the game may cause an emotional change, giving rise to nervousness for instance [44].

As no further significant differences were found, this means that it is necessary to increase the duration of the game with an appropriate session being deemed to last 30 minutes, in addition to also increasing the size of the sample in order to obtain more reliable results [45].

#### 5.3.2. Mann-Whitney Test

No differences were found between men and women, whereby it can be concluded that the sensors are ready for use both for men and women, there being no difference in terms of results nor in terms of the age of the participants [46].

#### 5.3.3. Correlations

The Spearman Rank-order correlation was chosen for the correlations made. Testing was carried out to ascertain whether there is any linear relationship between the values obtained from the PSS [47] and the values provided by the sensors (initial value, final value, maximum value, minimum value, and differences between maximum and minimum peaks).

**Figure 12:**
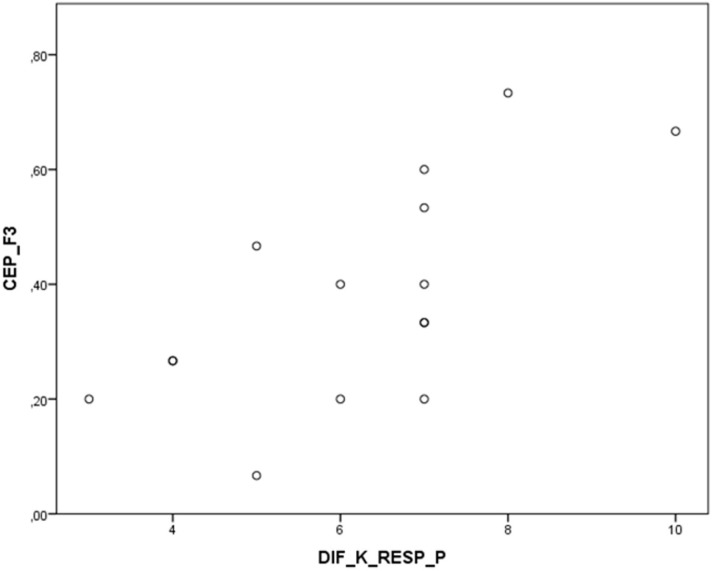
Correlation graph between factor 3 (PSS) and peaks in the breathing sensor.

A correlation was found between factor 3 (energy and joy) from the PSS and the difference between maximum and minimum peaks in the breathing sensor while participants were playing with the Kinect (r = 0.659, *p* = 0.008). This correlation can be seen in Figure 12, *i.e.*, the greater score in this factor, the heavier the participant’s breathing or the more nervous they are [48].

This correlation shows that the difference between maximum and minimum breathing levels when playing with the Kinect is closely correlated with the Perceived Stress Scale, specifically with the factor referring to the participant’s level of energy and joy. This factor included items such as feeling full of energy, feeling tired and happy, *etc.*, whereby the participant did not feel that they had sufficient energy and had not become so involved in the game, resulting in there not being so much difference maximum and minimum peaks.

Neither was any correlation found between factor 6 (fear and anxiety) from the PSS and the difference between maximum and minimum peaks in the breathing sensor with the Kinect (r = 0.712, *p* = 0.003). The breathing peaks obtained when playing with the Kinect were also correlated with the PSS questionnaire factor that measures fear and anxiety. As this is a positive correlation, this means that the greater the fear and anxiety that the participants experience, the more this is shown in the difference between maximum and minimum breathing levels.

There is also correlation between the total results obtained from the PSS questionnaire and the difference between maximum and minimum peaks in the breathing sensor, while also playing with the Kinect (r = 0.618, *p* = 0.014). This correlation shows that the greater the level of stress the person had, the more this translates into greater difference between maximum and minimum breathing levels during the game. This means that, the more stress experienced by the participant, the faster their pulse will beat at times when they are nervous during the game, as will have had to increase their pulse rate from the time they started the game and were in a calm state for this correlation to exist.

There is a correlation between factor 1 (tension, irritability and fatigue) from the PSS and the final value obtained from the breathing sensor when playing with the Eye Tracker (r = 0.596, *p* = 0.019).

If the correlation is a positive one, this means that the greater the participant’s tension, irritability and fatigue, the greater the breathing level the user will have at the end of the game. This makes sense, as if they normally experience stress in their daily life, they will also show this when playing and encountering difficulties during the game.

Correlation also exists between factor 1 (tension, irritability and fatigue) from the PSS and the final breathing level when playing with the Kinect (r = 0.682, *p* = 0.005). The same occurs in this correlation as with the previous one—Simply that there is a change in the mode of play that on this occasion is with the Kinect.

Another correlation existing is that found between factor 5 (satisfaction with self-fulfillment) from the PSS and the final breathing level when playing with the Kinect (r = 0.556, *p* = 0.032). There is also correlation between the total value obtained from the PSS questionnaire and the final breathing level when playing with the Kinect (r = 0.617, *p* = 0.014). There is correlation between factor 1 (tension, irritability and fatigue) from the PSS and the minimum cardio level when playing with the Eye Tracker (r = 0.515, *p* = 0.05).

#### 5.3.4. Summary Results

In summary, we found several significant results applying biofeedback techniques in this game. Having said that, we could find other results if the biofeedback sessions had larger and more numerous.

Firstly, the game received a good evaluation in terms of usability and satisfaction. Moreover the participants evaluated the sensors according to comfort, control and fitting. In Eye Tracker mode, EMG sensor received the greatest values, because the participants can control it better than the other sensors, it was comfortable and easy to fit on their body.

As regards the statistical results, when the participants are playing with the Eye Tracker, significant differences were found between the initial value just at the start of the game and the final value at the end of it from the GSR sensor. This means stress level change during the game, because person does not want to lose and is eager to progress in the game. But this change of stress level means that the player becomes nervous and the game status going against them. Therefore, the player can achieve control this level and improve their own play.

In addition to this, no differences were found between men and women, so sensors are ready for use by both men and women. The same for the age of the participants: no differences were found. Finally, the best correlation found, was between the energy and joy factor of PSS test, and the maximum and minimum peaks in the breathing sensor while participants were playing with the Kinect.

## 6. Conclusions

In this section we are going to gather the thoughts and conclusions obtained from this study as well as defining new challenges in the following phase of the study.

As for how usable the game is, a sufficiently high value was obtained, albeit one that could be improved on in terms of using wireless technology for greater comfort when playing. In particular, the GSR proved to be a highly-rated sensor that obtained very high averages in terms of how it is fitted and its comfort. As this is a sensor attached to two fingers, it hardly impedes any natural movement of a person’s arms. Therefore, this sensor is an obvious candidate to be retained as an end product.

The EMG sensor was positively rated by users in terms of its ability to control the game. As it is associated with a movement of the muscle, the natural movements that enable interaction during the game are respected as in the previous case, which is a positive factor for the players.

As regards the pulsometer and respirometer band, we should draw attention to the great variation between the maximum and minimum values associated with fitting the device on the person. There are players who found fitting extremely uncomfortable and others who were not so concerned about this. Taking into account the fact that it is fitted on the chest, we consider this to be a sensitive and fairly inaccessible area, whereby in the future its modified position on the body could be taken into account.

Use of the Kinect was a practice about which participants were aware, which is why in terms of preference, the Eye Tracker was the one that was chosen by most as the ideal one for the purpose of interacting with the game.

It was shown that the GSR associated with a person’s level of emotion and anxiety evidences higher values after they have played the game. This should be taken into account when prescribing this game to prevent people with some health problem from using it in case it gives rise to major changes in their emotional state, although it can be recommended for people who have been advised to pursue a life filled with strong emotions and surprises, *etc*.

Fortunately, it was ascertained that there were no differences either in sex or in age for the chosen group, and the expansion of this group in terms of ages and profiles will be considered in phase 2, including individuals with disability.

Breathing was significantly correlated with factors regarding energy, joy, fear, tension, irritability and fatigue from the PSS questionnaire. Thus, the breathing value will be the key to subsequent phases of research and also for its extended use in games among the population. Notwithstanding this, it would be interesting by way of an improvement to attach this value to a separate sensor from that of the pulsometer, as the latter proved uncomfortable to fit.

Lastly, sessions of longer duration will be provided as a continuation of this research insofar as the surprise factor of initial interaction with the game during steady state operation will be disregarded. The experience gained from this biofeedback research involving interaction with the game has enabled us to ascertain the suitability of the set of sensors chosen in terms of the requirements needed to interact with a game. Beyond the field of leisure, the authors consider this to be an interesting fact for rehabilitation therapy based on biofeedback techniques.

## Supplementary Files

Supplementary File 1
