# Supplementary material for: Game Design to Measure Reflexes and Attention Based on Biofeedback Multi-Sensor Interaction
Source: Sensors (Basel). 2015 Mar 17;15(3):6520–48. doi: 10.3390/s150306520 (PMC4435215; doi:10.3390/s150306520)
Supplement: Supplementary File 1 [file sensors-15-06520-s001.pdf]

OPEN ACCESS

sensors

ISSN 1424-8220

www.mdpi.com/journal/sensors

Supplementary Information

Game Design to Measure Reflexes and Attention Based on Biofeedback Multi-Sensor Interaction. *Sensors* 2015, *15*, 6520-6548

Inigo de Loyola Ortiz-Vigon Uriarte *, Begonya Garcia-Zapirain and Yolanda Garcia-Chimeno

Deusto-Tech-LIFE Department, University of Deusto, Bilbao 48007, Spain;
E-Mails: mbgarciazapi@deusto.es (B.G.-Z.); yolanda.garcia@deusto.es (Y.G.-C.)

* Author to whom correspondence should be addressed; E-Mail: inigo.ovu@gmail.com;
Tel.: +34-944-139-000 (ext. 2980); Fax: +34-944-456-817.

---

1. Socio-Demographic Questionnaire

Age: ________

Gender: Male Female

Do you have any visual conditions? Yes No

If yes indicate which: ____________________________

Do you use glasses? Yes No

Do you use contact lenses? Yes No

Do you play videogames? Yes No

How often?

At some time a month At Weekends

At least three or four days per week Every day

Which type of videogames you play the most? (Choose one option)

Arcade games (platforms games, maze games) Tetris, Mario Bros…

Simulation games (instrumental games, situational games, sports games) FIFA, Gran Turismo…

Strategy games (graphic adventures, role games, war games) Age of Empires, GTA…

Board games (cards, cultural games) Solitaire, Poker

2. Perceived Stress Scale Questionnaire

For each question, **mark with an X** the box that best describes you **in the last month**:

	Never	Sometimes	Often	Usually
1. You feel rested.	1	2	3	4
2. You feel that too many demands are being made on you.	1	2	3	4
3. You are irritable or grouchy.	1	2	3	4
4. You have too many things to do.	1	2	3	4
5. You feel lonely or isolated.	1	2	3	4
6. You find yourself in situations of conflict.	1	2	3	4
7. You feel you're doing things that are really like.	1	2	3	4
8. You feel tired.	1	2	3	4
9. You fear you may not manage to attain your goals.	1	2	3	4
10. You feel calm.	1	2	3	4
11. You have too many decisions to make.	1	2	3	4
12. You feel frustrated.	1	2	3	4
13. You are full of energy.	1	2	3	4
14. You feel tense.	1	2	3	4
15. Your problems seem to be piling up.	1	2	3	4
16. You feel you're in a hurry.	1	2	3	4
17. You feel safe and protected.	1	2	3	4
18. Have a many worries.	1	2	3	4
19. You are under pressure from other people.	1	2	3	4
20. You feel discouraged.	1	2	3	4
21. You enjoy yourself.	1	2	3	4
22. You are afraid of the future.	1	2	3	4
23. You feel you're doing things because you have to, not because you want.	1	2	3	4
24. You feel criticized or judged.	1	2	3	4
25. You feel lighthearted.	1	2	3	4
26. You feel mentally exhausted.	1	2	3	4
27. You have trouble relaxing.	1	2	3	4
28. You feel loaded down with responsibility.	1	2	3	4
29. Have enough time for yourself.	1	2	3	4
30. You feel under pressure from deadlines.	1	2	3	4

3. System Usability Scale Questionnaire

For each question, **mark with an X** the box that best describes your reaction to this tool:

	Totally Disagree	Disagree	Undecided	Agree	Totally Agree
I think that I would like to use this system frequently	1	2	3	4	5
I found the system unnecessarily complex	1	2	3	4	5
I thought the system was easy to use	1	2	3	4	5
I think that I would need the support of a technical person to be able to use this system	1	2	3	4	5
I found the various functions in this system were well integrated	1	2	3	4	5
I thought there was too much inconsistency in this system	1	2	3	4	5
I would imagine that most people would learn to use this system very quickly	1	2	3	4	5
I found the system very cumbersome to use	1	2	3	4	5
I felt very confident using the system	1	2	3	4	5
I needed to learn a lot of things before I could get going with this system	1	2	3	4	5

4. Preferences Questionnaire

Game with Eye tracker

For each question, **mark with an X** the box that best describes your reaction to this tool (**where 1 means totally disagree and 5 means totally agree**):

**Sensors in the chest**					
Is easy to put on the sensor	1	2	3	4	5
During the game is confortable	1	2	3	4	5
I feel that I can control the game by means of the pulse/ heart rate	1	2	3	4	5
I feel that I can control the game by means of the respiration	1	2	3	4	5
**GSR: sensors in the fingers**					
Is easy to put on the sensor	1	2	3	4	5
During the game is confortable	1	2	3	4	5
I feel that I can control the game by means of the skin resistance	1	2	3	4	5
**EMG: arm sensor**					
Is easy to put on the sensor	1	2	3	4	5
During the game is confortable	1	2	3	4	5
I feel that I can control the game by means of the muscular response	1	2	3	4	5

Organize the sensors (Cardio, respiration, skin resistance, muscular response) depending on preference:

Preference	Sensor	Reason
1		
2		
3		
4		

Game with Kinect

For each question, **mark with an X** the box that best describes your reaction to this tool (**where 1 means totally disagree and 5 means totally agree**):

**Sensors in the chest**					
Is easy to put on the sensor	1	2	3	4	5
During the game is confortable	1	2	3	4	5
I feel that I can control the game by means of the pulse/ heart rate	1	2	3	4	5
I feel that I can control the game by means of the respiration	1	2	3	4	5

Organize the sensors (Cardio, respiration) depending on preference:

Preference	Sensor	Reason
1		
2		

Game

I liked playing with the EyeTracker	1	2	3	4	5
I liked playing with Kinect	1	2	3	4	5

Which one did you like more? Why?

____

____

____

Comments:____

____

____

____

© 2015 by the authors; licensee MDPI, Basel, Switzerland. This article is an open access article distributed under the terms and conditions of the Creative Commons Attribution license (http://creativecommons.org/licenses/by/4.0/).
